# Supplementary material for: MetaRibo-Seq measures translation in microbiomes
Source: Nat Commun. 2020 Jun 29;11:3268. doi: 10.1038/s41467-020-17081-z (PMC7324362; doi:10.1038/s41467-020-17081-z)
Supplement: Supplementary file 10 — Supplementary Data 7 [file 41467_2020_17081_MOESM10_ESM.zip › File2/Confidence_VeryHigh_Taxonomy/138009_out.krona.html]

Javascript must be enabled to view this page.

members
magnitude
magnitudeUnassigned
count
unassigned
taxon
rank

138009\_out

4


SRS056896\_contig\_number\_contig-100\_8120.54078SRS144014\_contig\_number\_contig-100\_18148.46283
2

2
2
superkingdom

1239
phylum
1

1
class
186801

1
186802
order

1
31979
family

1485
genus
1

1
1042156

SRS097918\_contig\_number\_contig-100\_3025.65160
species

1
32066
phylum

1
203490
class

1
order
203491

family
1129771
1

1
genus
34104

1

SRS017808\_contig\_number\_9677
34105
species
